# Metformin-Induced Vitamin B12 Deficiency in Patients With Type-2 Diabetes Mellitus

**DOI:** 10.7759/cureus.47771

**Published:** 2023-10-26

**Authors:** Aakriti Tiwari, Rakshit Kumar Singh, Prasiddhi D Satone, Revat J Meshram

**Affiliations:** 1 Medicine, Jawaharlal Nehru Medical College, Datta Meghe Institute of Higher Education and Research, Wardha, IND; 2 Paediatrics, Jawaharlal Nehru Medical College, Datta Meghe Institute of Higher Education and Research, Wardha, IND

**Keywords:** hyperglycemia, methylcobalamin, diabetes mellitus, type-2 diabetes mellitus, malabsorption, anemia, neuropathy, metformin, vitamin b12 deficiency

## Abstract

Diabetes mellitus (DM) is the most common metabolic disease worldwide. Hence, the prevalence of the disease continues to increase across the globe. Metformin is used as a first-line oral hypoglycemic drug to keep control of type-2 DM (T2DM) in adults. Diabetic patients on metformin have been largely seen to be suffering from a deficiency of vitamin B12. It is a water-soluble vitamin mainly obtained from animal food like meat. At the basic cell level, it acts as a cofactor for enzymes essential for DNA synthesis and neuroprotection. As a result, vitamin B12 deficiency can show clinical effects such as progressive demyelination, peripheral neuropathy and haematological abnormalities (such as macrocytic anaemia and neutrophil hypersegmentation). Various studies also show a relation between vitamin B12 insufficiency and metformin-treated T2DM patients as decreased absorption of vitamin B12. There could be a severe complication of vitamin B12 deficiency in T2DM patients. The use of proton pump inhibitors, gastric bypass surgery, older patients and patients with a higher red blood cell turnover are factors that hasten the depletion of vitamin B12 reserves in the liver. Methylmalonic acid and homocysteine levels can be measured to identify vitamin B12 insufficiency at its early stage if blood vitamin B12 levels are borderline. The action of metformin on vitamin B12 absorption and its potential mechanisms of inhibition will be the main topics of discussion in this review. The review will also discuss how vitamin B12 deficiencies in T2DM patients using metformin affect their clinical results.

## Introduction and background

Type-2 diabetes mellitus (T2DM) is a metabolic condition that is increasing public health awareness. Numerous systemic macrovascular and microvascular problems are linked to the condition. T2DM can impair a person's cardiovascular, neurological, and renal systems in addition to having a high death rate [1]. The most frequent consequence, which can develop in up to 50% of people, is diabetic peripheral neuropathy (DPN) [1]. Every day that goes by, there is a global trend toward an increase in the prevalence of diabetes. The management strategy for T2DM often starts with one anti-glycemic medication, which is usually a biguanide, metformin [2]. It decreases glucose synthesis from the liver while somewhat enhancing peripheral glucose utilization. Through organic cation transporters, metformin enters cells and activates the adenosine monophosphate (AMP) dependent protein kinase. Metformin is the most effective drug which also lowers blood glucose, improves lipid profiles, and causes mild weight reduction [3].

Nonetheless, some disadvantages of metformin use are noted in diabetics, including metformin-induced vitamin B12 deficiency [4]. The mechanism behind it can be either directly reducing the vitamin B12 absorption or altering the motility of the small intestine [5]. According to reports, 14% to 30% people on long-term metformin have lower level of vitamin B12 in blood, and 30% develop vitamin B12 malabsorption [6]. After ingestion, vitamin B12 is released by undergoing proteolytic breakdown of its carrier proteins present in food at acidic pH of the stomach. In the stomach, vitamin B12 attaches to haptocorrin, a cobalamin binding protein [7]. Haptocorrin is produced and released by salivary glands of the oral cavity. Vitamin B12 is protected from breakdown in the acidic environment of stomach by haptocorrin-vitamin B12 complex. After reaching duodenum, haptocorrin-vitamin B12 complex undergoes cleavage due to the changes in pH and breakdown of haptocorrin by the pancreatic proteases, which leads to the release of vitamin B12 in its free form. A complex of intrinsic factor (IF) and vitamin B12 is formed in the duodenum after the release of IF from the parietal cells of the stomach and its attachment to free vitamin B12 [8]. The newly formed IF-vitamin B12 complex is subsequently bound by the cubilin receptor on the distal end of the ileum in a calcium-dependent manner, resulting in vitamin B12 absorption by endocytosis. Internalization of the IF and vitamin B12 complex results in the release of the complex from its receptor and passage into the bloodstream [9]. Transcobalamin gives the site of attachment for 20%-25% of vitamin B12. Holotranscobalamin (HoloTC) is another name for the transcobalamin-vitamin B12 complex, which is the active form of cobalamin that enables the absorption of vitamin B12 into the cells through specific transcobalamin receptors. The liver stores 70%-80% of vitamin B12 bound to haptocorrin. A part of vitamin B12 is excreted in bile and circulated throughout the liver's enterohepatic system [10].

In the cells, vitamin B12 participates in two reactions, firstly in the cytosolic mechanism mediated by the enzyme methionine synthase for which it acts as a cofactor. Here it aids in the transmission of a methyl group to homocysteine from the reaction, 5-methyltetrahydrofolate to tetrahydrofolate, to create 2-amino-4-(methylthio)butanoic acid (methionine). Additionally, as an intermediary step in fatty acid oxidation and ketogenic amino acid catabolism. Here it helps convert methylmalonyl coenzyme-A (CoA) to succinyl CoA catalyzed by the enzyme methylmalonyl-CoA mutase. Vitamin B12 deficiency halts this process, which causes methyl malonyl CoA and homocysteine to accumulate, which consecutively gets converted into methylmalonic acid. Hence, the functional markers for vitamin B12 insufficiency include increased serum or plasma homocysteine and methylmalonic acid levels [11].

## Review

Methodology

**Figure 1:**
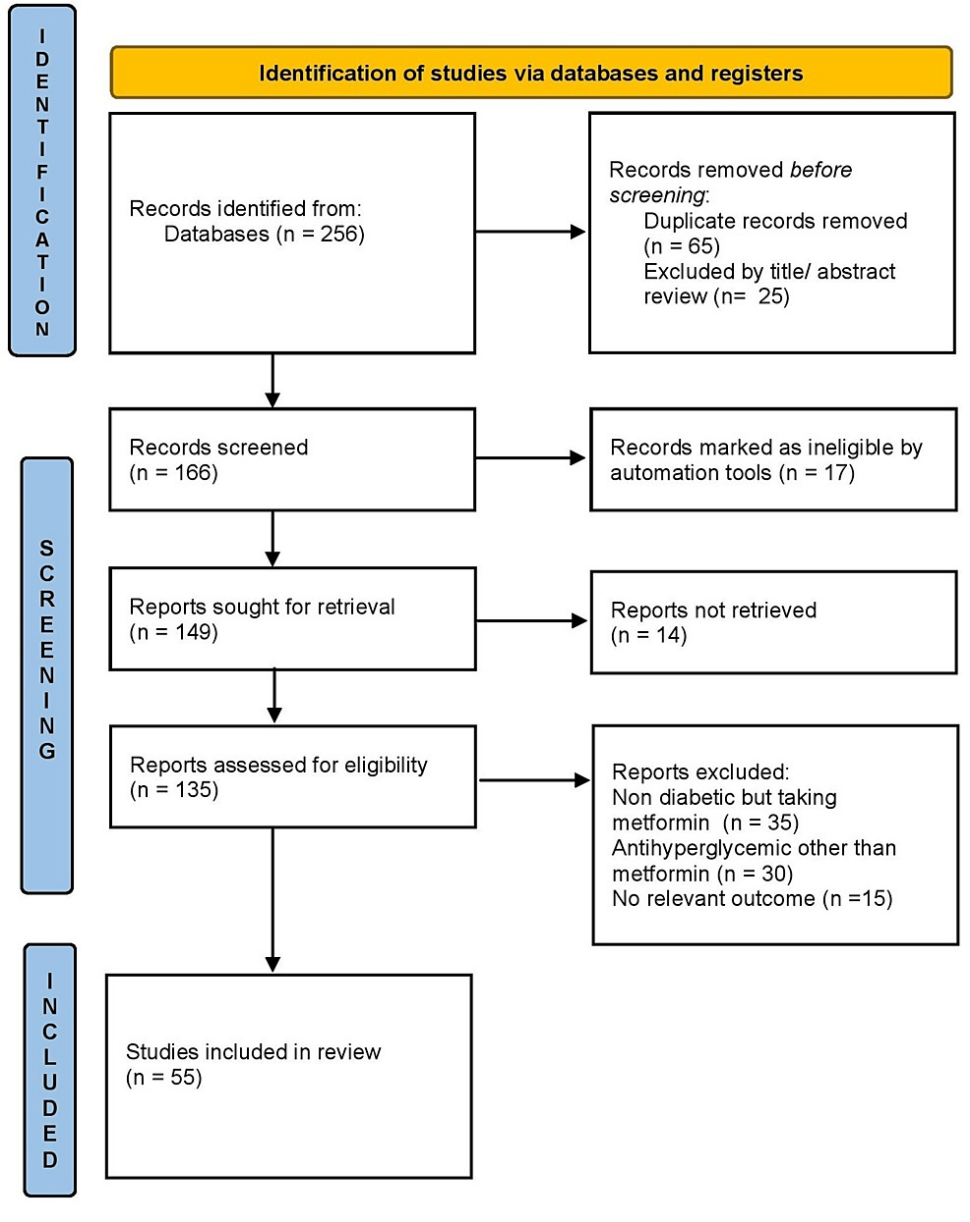
PRISMA diagram Authors' creation PRISMA: Preferred Reporting Items for Systematic Reviews and Meta-Analyses

The search methodology followed a comprehensive approach to identify relevant studies for the review. The process involved searching various databases, defining inclusion and exclusion criteria, screening articles, and selecting the final studies for the review. A comprehensive search was conducted in electronic databases, including PubMed. These databases were chosen to ensure broad coverage of relevant literature. The search included studies published from the inception of the databases up to 30 August 2023, with no specific date restrictions. The search strategy incorporated a combination of key terms and Medical Subject Heading (MeSH) terms related to metformin, type-2 diabetes mellitus, pathophysiology, clinical manifestation, and, management of vitamin B12 deficiency. The key terms included are type-2 diabetes mellitus, malabsorption, anemia, neuropathy, metformin, vitamin b12 deficiency, diabetes mellitus, hyperglycemia, methylcobalamin, IF, and relevant synonyms. Only articles published in English were considered. Case reports and editorials were excluded from the review. The initial screening involved reviewing the titles and abstracts of the identified articles based on the inclusion and exclusion criteria. Full-text articles were obtained for the potentially relevant studies, and further screening was conducted to select the final articles for the review. A total of 54 articles met the inclusion criteria and were included in the final review. The following Preferred Reporting Items for Systematic Reviews and Meta-Analyses (PRISMA) flow diagram provides a visual representation of the search methodology, showing the number of articles identified, screened, and included in the final review (Figure 1).

Pathophysiology

**Figure 2:**
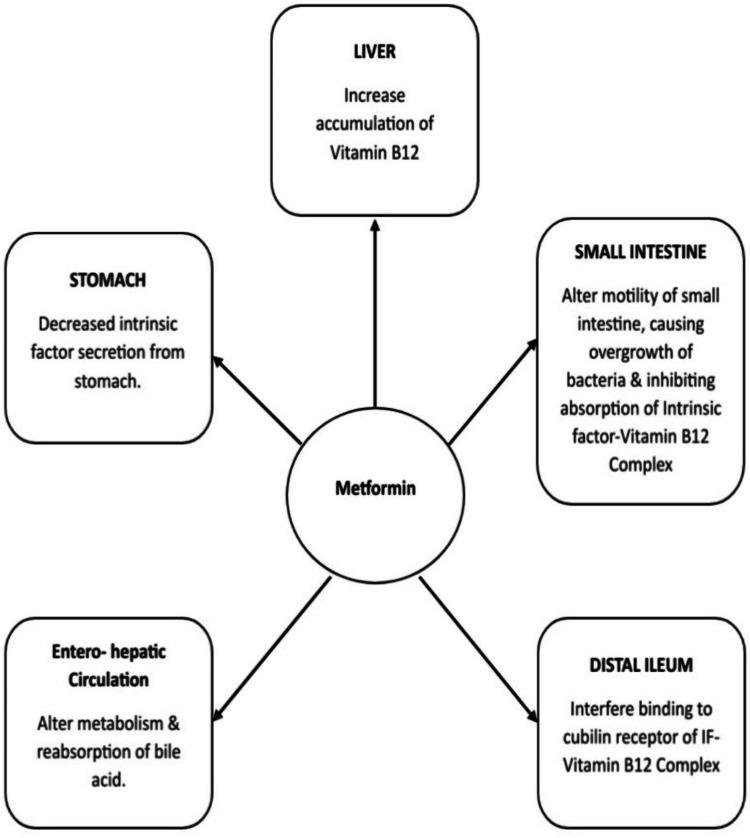
Metformin-induced vitamin B12 deficiency Authors' creation IF: Intrinsic factor

The proposed mechanisms involved in vitamin B12 deficiency induced due to long-term use of metformin are not very familiar until now [12]. However, the main mechanisms causing the deficiency are the alteration of absorption and metabolism of vitamin B12. The various mechanisms (as seen in Figure 2) can be metformin travels to the liver and causes increased accumulation of vitamin B12, leading to decreased distribution in tissues and altered metabolism of vitamin B12 [13]; interference with the binding of IF-vitamin B12 complex to the cubilin receptors on the intestinal villi and enterocytes at the level of the ileum [14]; reduced secretion of IF by stomach parietal cells [15]; impairment of enterohepatic recycling of vitamin B12 due to alteration in metabolism and reabsorption of bile acid [16]; and reduced motility of the small intestine results in bacterial overgrowth, preventing the IF-vitamin B12 combination from being absorbed in the distal ileum [17]. 

Recently, it was proposed that metformin only reduces the amount of circulating vitamin B12 and has no impact on the levels of intracellular vitamins. Target cells requiring vitamin B12 are reached by a complicated pathway including numerous proteins and receptors. Understanding this multi-step course is necessary to comprehend the complex nature of vitamin B12 insufficiency and the issues accompanying its diagnosis [18]. Then the vitamin is found in the bloodstream attached to either transcobalamin-I (TC-I) or transcobalamin-II (TC-II). 20-30% of the total amount of circulating vitamin B12 is thought to be bound to TC-II protein. Newly absorbed vitamins are bound by the protein and transported to the target tissues where they are absorbed via a receptor-mediated internalization process. Measuring TC-II level bound to vitamin B12 is used as a diagnostic method to assess vitamin B12 status. 70-80% of the circulating vitamin B12 is bound by TC-I, preventing the loss of the free, unnecessary fraction [19].

Clinical manifestations and assessment of deficiency

Usually, metformin is widely used in long-term diabetes control. The clinical features of metformin-induced vitamin B12 deficiency include hematological manifestations in the form of megaloblastic anemia and neurological complications, i.e., peripheral neuropathy, degeneration of the spinal cord, and decline of cognition [20]. Cognitive decline may become apparent or develop into dementia in people with macrocytic anemia because it affects the production and shape of white blood and red blood cells. Numerous hematologic and neuronal consequences, as well as the four biomarkers of blood, which are total vitamin B12 level, HoloTC, homocysteine, and methylmalonic acid, are markers of vitamin B12 status. The problem is that not any one of these indicators individually has the optimum specificity and sensitivity for vitamin B12 insufficiency [21]. The respective cut-off values for serum total vitamin B12, HoloTC indicating deficiency is <148 picomol/L, <35-40 picomol/L [19]. The respective cut-off values for elevated homocysteine and methylmalonic acid are >12-15 µmol/L and >370 nmol/L [22]. The idea is that measuring two out of the four biomarkers of blood to determine the status of vitamin B12 would increase the specificity and sensitivity for diagnosis compared to measuring only one biomarker [23]. Recently, a method has been devised that uses all four biomarkers concurrently to provide a single value known as combined vitamin B12 [24]. The cut-off range of combined vitamin B12 has been determined to correlate to several classes of the status of vitamin B12 and are adequate -0.5 to 1.5, deficient -2.5 to -1.5, and elevated >1.5. Initial evaluations of the use of combined B12 in assessing the status of vitamin B12 have shown that it has more tremendous diagnostic potential than any one of the other four biomarkers by itself [25,26]. The frequency of vitamin B12 insufficiency differs with populations. Globally, several countries have varied dietary practices and eating habits. Regular vitamin B12 supplementation cannot reverse the biochemical B12 reduction in diabetics receiving metformin, according to a national survey [27]. There was a solid dose-dependent relationship between the prevalence of vitamin B12 deficiency and metformin use, and a connection was discovered relating to the period of metformin use. Recent cross-sectional studies have shown that more than a thousand-milligram dosages have shown a correlation with vitamin B12 insufficiency that is independent [28]. 

High-risk group for developing vitamin B12 deficiency

Apart from individuals suffering from T2DM, numerous conditions are substantially involved with vitamin B12 insufficiency, which will be discussed in this section. Elderly people, pregnant women, and particular ethnic and racial groups are more likely to develop vitamin B12 insufficiency. Older people have more chances of vitamin B12 deficiencies, especially those over 65 years of age, with a cobalamin insufficiency prevalence of 10%-15% [29,30]. Many conditions lead to vitamin B12 deficiency, and the underlying process behind them has been mentioned in Table 1 [31,32]. For an accurate differential diagnosis, such conditions should be quickly recognized in patients taking metformin with vitamin B12 insufficiency. Because cobalamin may be transferred from the maternal to the fetal and newborn in the phase of pregnancy, it can also affect the mother's vitamin B12 level. Prevalence estimates of vitamin B12 insufficiency during pregnancy have been found to range from less than 10% in Canada to more than 70% in India [33,34]. According to a cross-sectional survey conducted in New Zealand, the Pacific Islands and certain Southeast Asian countries, including Indonesia, Malaysia, Vietnam and the Philippines, endured the highest levels of vitamin B12, while South Asians, as well as those with ancestry in India are at maximum risk of decreased vitamin B12 status [35]. Another study indicated that South Asians have a more significant occurrence of vitamin B12 insufficiency than the overall population [36]. Vitamin B12 (cobalamin) deficiencies during infancy negatively affect the growing brain, whereas folate deficiencies during the periconceptional phase lead to neural tube malformations. Deficiencies in both vitamins are related to an increased risk of depression in adults [36]. In diabetic individuals with neuronal involvement, metformin-induced vitamin B12 deficiency can worsen nerve damage, leading to neuropathy caused by cobalamin deficiency [37]. Furthermore, moderate to severe peripheral neuropathy associated with diabetes occurred much more frequently in metformin-treated individuals [37].

**Table 1:** Conditions leading to vitamin B12 deficiency and its underlying process IF: Intrinsic factor

Conditions leading to vitamin B12 deficiency	Underlying process
Chronic alcoholism, strict vegan diet, malnutrition	Decreased intake of food that contains vitamin B12 in the diet
Elderly age group	Decreased absorption of vitamin B12 and reduced intake in diet causing deficiency
Gastric bypass surgery, total or partial gastrectomy, weight loss surgery and chronic gastritis	Inadequate secretion of IF
Long-term (more than a year) use of medications that control stomach acid output or change the pH of the stomach, such as antacids, proton pump inhibitors, and histamine receptor antagonists	These drugs cause inadequate pepsin activity and insufficient hydrochloric acid, which prevent vitamin B12 from being adequately liberated from the food matrix
Long-term metformin usage	The primary mechanism by which metformin ensues in a vitamin B12 deficit is unspecific. It might be one of the following: (1) Increased accumulation of vitamin B12 in the hepatocytes; (2) Interfering with the IF-vitamin B12 complex's ability to bind to the cubilin receptor in a calcium-dependent manner; (3) Reduced secretion of IF by the parietal cells present in the stomach; (4) Decreased enterohepatic recycling of vitamin B12; (5) Diminished motility of the small intestine leading to decreased retention of IF-vitamin B12 complex from the distal ileum
Exocrine pancreatic disease	Decreased activity of pancreatic enzymes leads to reduced proteolytic breakdown of haptocorrin. Additionally, the distal ileum does not allow for the absorption of vitamin B12, which has already been bonded to haptocorrin
Parasitic infestation of the intestine (by protozoan or tapeworm)	Decreased retention of vitamin B12 due to its trapping by the parasitic organisms

Benefits of metformin in systemic diseases

According to recent animal research, metformin has neuroprotective and anti-neuropathic properties that are separate from its euglycemic impact. In mice, metformin guards against the neuropathic discomfort and numbness brought on by chemotherapy [38]. Metformin has also been shown in studies on animals to eliminate pain brought on by sensory neuron stimulation, in addition to treating neuropathic allodynia, protecting against alcohol-induced apoptosis of neurons, and increasing neurogenesis. Additionally, it prevented neuronal death in cortical neurons and had neuroprotective benefits for Parkinson's patients [39]. Metformin significantly benefits T2DM, cancerous diseases, neurodegenerative and cardiac diseases, aging, reproductive system diseases, Covid-19, and Down syndrome. Metformin also has side effects, such as gastrointestinal issues, lactic acidosis, neurodegenerative disease, and vitamin B12 deficiency [40]. The use of metformin in a regimen with other antihyperglycemic medications, such as sodium-dependent glucose transporters-2 inhibitors, dipeptidyl peptidase-4 inhibitors, glibenclamide, glucagon-like peptide-1 receptor agonists, is more effective than metformin alone in lowering blood glucose levels [41]. In newly diagnosed T2DM patients, metformin medication significantly reduces the risk of cardiovascular events compared to standard therapy (diet management), and a 39% risk reduction of myocardial infarction was documented [42]. Worldwide, polycystic ovarian syndrome (PCOS) affects around 5-20% of reproductive-aged women, and the majority of cases are marked by elevated androgens, insulin resistance, acanthosis nigricans, ovulatory dysfunction, and other symptoms. Researchers discovered that metformin therapy increased the chances of conception for PCOS-affected women [43]. Even though aging is an inevitable phase of life, metformin was chosen to be studied for its impact on aging benefits due to its anti-inflammatory qualities and restoration of redox effects. Metformin has been shown in several epidemiological studies to slow the aging process and lower mortality from all causes of age-related disorders [44]. Metformin may be able to lower cancer-related mortality as well as cancer risk, according to epidemiological research [45]. Metformin slightly reduces the risk of adenomas, pancreatic duct adenocarcinoma, and endometrial cancers [45]. Most active clinical studies of cancer treatment use metformin in conjunction with recognized cytotoxic chemotherapy [46]. A drug's safety must be thoroughly considered, in addition to its efficacy and advantages. Because of its pleiotropic method of action, metformin not only treats several ailments but also has several adverse effects. Gastrointestinal issues are frequently the adverse effect of metformin medication that is most commonly observed, affecting 2-63% of T2DM patients, including diarrhea, nausea, vomiting, pain in the abdomen, and flatulence [47].

Management of vitamin B12 deficiency 

A frequent illness, vitamin B12 insufficiency can manifest clinically as non-specific symptoms or, in more severe situations, as neurological or hematopoietic abnormalities. Although pernicious anemia was once the primary cause, this disorder is now only seen in a few cases, and food-bound cobalamin malabsorption is responsible for vitamin B12 insufficiency [48]. First and foremost, it is essential to prevent the emergence of frank vitamin B12 deficiency in those who are at increased risk for it. When it does, it is essential to provide an appropriate replacement [10]. With this strategy, it is possible to avoid the clinical effects of vitamin B12 insufficiency, such as megaloblastic anemia, and neurological manifestations like peripheral neuropathy. Patient without neurological damage requires hydroxocobalamin 1 mg on alternating days for 14 days, followed by three-monthly injections of hydroxocobalamin 1mg. If due to pernicious anemia, this regimen should be taken for the rest of one's life [49]. If there is another underlying reason for the deficit, therapy should continue until persistent improvement in hematological indices is observed. When there are neurological symptoms, the dosage is continued until no more clinical relief is observed, after which two monthly injections are administered [49]. Recommend referral of pregnant females to a secondary care center if severe neurological manifestations occur. It is frequently beneficial to treat underlying issues, such as antibiotics for bacterial overgrowth, and to stop using the offending medications [50]. The patient's reaction to therapy must be monitored once the diagnosis of vitamin B12 deficit has been determined and a treatment plan has been started. If severe anemia and vitamin B12 deficiency are linked, correcting the deficient condition should result in a noticeable reticulocyte count increase in seven to fourteen days [51]. The unfavorable effects of newborns with vitamin B12-associated neuropathy can be reduced with cobalamin therapy [52]. Numerous case studies involving young children found that vitamin B12 treatment improved their hematological status and quickly improved symptoms like lethargy and reduced activity [53]. Cobalamin deficiency may hinder early development by causing problems with myelination, dendritic formation, or inflammation. There are no current guidelines regarding the preventive use of oral vitamin B12 for individuals on metformin [54]. The various clinical studies done till date regarding the measurement of the prevalence of vitamin B12 deficiency, their diagnostic cut-off point and other sample and study characteristics are mentioned in Table 2 [55]. 

**Table 2:** Clinical studies that measured the prevalence of metformin-induced vitamin B12 deficiency, their diagnostic cut-off points and other sample and study characteristics NHANES: National Health and Nutrition Examination Survey

Study	Obtained prevalence	Cut-off point of vitamin B12 (pmol/L)	Study setting
Reinstatler et al. [19]	5.80%	<148	NHANES, United States
Liu et al. [27]	29%	<150	Geriatric outpatient clinic, Hong Kong
Ahmed et al. [2]	28.10%	<150	Outpatient diabetes clinics of two
			tertiary hospitals, South Africa
Yakubu et al. [28]	32.10%	<135	Diabetic clinic of the Effia Nkwanta Regional Hospital, Takoradi, Ghana
Miller et al. [26]	6.50%	<148	Latino ancestry residing in Sacramento, California
Pennypacker et al. [29]	14.50%	<148	University Health Sciences Center, Denver, Colorado

## Conclusions

In conclusion, the widespread and often indispensable use of metformin in the treatment of T2DM has shed light on a significant yet underappreciated consequence, the potential to induce vitamin B12 deficiency. This article has delved into the intricate relationship between metformin and vitamin B12, highlighting the mechanisms by which the drug may disrupt the absorption and utilization of this essential nutrient. The evidence presented underscores the importance of vigilance among healthcare professionals and patients alike. Routine monitoring of vitamin B12, especially metformin users, for extended periods, is imperative to detect and address the deficiency in its early stages. Routine supplementation or dietary adjustments can mitigate the risk of adverse effects on hematological, neurological, and metabolic health. Furthermore, this discussion prompts a broader reflection on the complex interplay between pharmaceutical interventions and nutrient balance. While metformin undoubtedly offers invaluable benefits in diabetes management, its potential impact on vitamin B12 absorption emphasizes the need for a holistic approach to patient care. Practitioners should consider personalized treatment plans that account for the potential nutrient deficiencies associated with certain medications. Looking ahead, continuing research is necessary to develop our knowledge about metformin-vitamin B12 interaction and find creative solutions to reduce the dangers of deficiencies. By fostering collaboration between healthcare providers, researchers, and pharmaceutical developers, we can optimize the benefits of metformin therapy while minimizing its unintended consequences.

## References

[1] (2023). Diabetes statistics. https://www.niddk.nih.gov/health-information/health-statistics/diabetes-statistics.

[2] Ahmed MA, Muntingh GL, Rheeder P (2017). Perspectives on peripheral neuropathy as a consequence of metformin-induced vitamin B12 deficiency in T2DM. Int J Endocrinol.

[3] Khattar D, Khaliq F, Vaney N, Madhu SV (2016). Is metformin-induced vitamin B12 deficiency responsible for cognitive decline in type 2 diabetes?. Indian J Psychol Med.

[4] Diamanti-Kandarakis E, Christakou CD, Kandaraki E, Economou FN (2010). Metformin: an old medication of new fashion: evolving new molecular mechanisms and clinical implications in polycystic ovary syndrome. Eur J Endocrinol.

[5] Ting RZ, Szeto CC, Chan MH, Ma KK, Chow KM (2006). Risk factors of vitamin B(12) deficiency in patients receiving metformin. Arch Intern Med.

[6] Tesfaye S, Boulton AJ, Dyck PJ (2010). Diabetic neuropathies: update on definitions, diagnostic criteria, estimation of severity, and treatments. Diabetes Care.

[7] Kakarlapudi Y, Kondabolu SK, Tehseen Z (2022). Effect of metformin on vitamin B12 deficiency in patients with type 2 diabetes mellitus and factors associated with it: a meta-analysis. Cureus.

[8] Infante M, Leoni M, Caprio M, Fabbri A (2021). Long-term metformin therapy and vitamin B12 deficiency: an association to bear in mind. World J Diabetes.

[9] Beedholm-Ebsen R, van de Wetering K, Hardlei T, Nexø E, Borst P, Moestrup SK (2010). Identification of multidrug resistance protein 1 (MRP1/ABCC1) as a molecular gate for cellular export of cobalamin. Blood.

[10] Green R, Allen LH, Bjørke-Monsen AL (2017). Vitamin B(12) deficiency. Nat Rev Dis Primers.

[11] Mazokopakis EE, Starakis IK (2012). Recommendations for diagnosis and management of metformin-induced vitamin B12 (Cbl) deficiency. Diabetes Res Clin Pract.

[12] Greibe E, Miller JW, Foutouhi SH, Green R, Nexo E (2013). Metformin increases liver accumulation of vitamin B12-an experimental study in rats. Biochimie.

[13] Adams JF, Clark JS, Ireland JT, Kesson CM, Watson WS (1983). Malabsorption of vitamin B12 and intrinsic factor secretion during biguanide therapy. Diabetologia.

[14] Caspary WF, Zavada I, Reimold W, Deuticke U, Emrich D, Willms B (1977). Alteration of bile acid metabolism and vitamin-B12-absorption in diabetics on biguanides. Diabetologia.

[15] Andrès E, Noel E, Goichot B (2002). Metformin-associated vitamin B12 deficiency. Arch Intern Med.

[16] Bauman WA, Shaw S, Jayatilleke E, Spungen AM, Herbert V (2000). Increased intake of calcium reverses vitamin B12 malabsorption induced by metformin. Diabetes Care.

[17] Ito H, Ishida H, Takeuchi Y, Antoku S, Abe M, Mifune M, Togane M (2010). Long-term effect of metformin on blood glucose control in non-obese patients with type 2 diabetes mellitus. Nutr Metab (Lond).

[18] Obeid R (2014). Metformin causing vitamin B12 deficiency: a guilty verdict without sufficient evidence. Diabetes Care.

[19] Reinstatler L, Qi YP, Williamson RS, Garn JV, Oakley GP Jr (2012). Association of biochemical B₁₂ deficiency with metformin therapy and vitamin B₁₂ supplements: the National Health and Nutrition Examination Survey, 1999-2006. Diabetes Care.

[20] Zhang C, Luo J, Yuan C, Ding D (2020). Vitamin B12, B6, or folate and cognitive function in community-dwelling older adults: a systematic review and meta-analysis. J Alzheimers Dis.

[21] Fedosov SN, Brito A, Miller JW, Green R, Allen LH (2015). Combined indicator of vitamin B12 status: modification for missing biomarkers and folate status and recommendations for revised cut-points. Clin Chem Lab Med.

[22] Fedosov SN (2013). Biochemical markers of vitamin B12 deficiency combined in one diagnostic parameter: the age-dependence and association with cognitive function and blood hemoglobin. Clin Chim Acta.

[23] Hannibal L, Lysne V, Bjørke-Monsen AL (2016). Biomarkers and algorithms for the diagnosis of vitamin B12 deficiency. Front Mol Biosci.

[24] Fedosov SN (2010). Metabolic signs of vitamin B(12) deficiency in humans: computational model and its implications for diagnostics. Metabolism.

[25] Brito A, Verdugo R, Hertrampf E (2016). Vitamin B-12 treatment of asymptomatic, deficient, elderly Chileans improves conductivity in myelinated peripheral nerves, but high serum folate impairs vitamin B-12 status response assessed by the combined indicator of vitamin B-12 status. Am J Clin Nutr.

[26] Miller JW, Garrod MG, Rockwood AL, Kushnir MM, Allen LH, Haan MN, Green R (2006). Measurement of total vitamin B12 and holotranscobalamin, singly and in combination, in screening for metabolic vitamin B12 deficiency. Clin Chem.

[27] Liu Q, Li S, Quan H, Li J (2014). Vitamin B12 status in metformin treated patients: systematic review. PLoS One.

[28] Yakubu M, Laing EF, Nsiah P (2019). Vitamin B12 deficiency in type 2 diabetic patients on metformin: a cross-sectional study from south-western part of Ghana. Alex J Med.

[29] Pennypacker LC, Allen RH, Kelly JP (1992). High prevalence of cobalamin deficiency in elderly outpatients. J Am Geriatr Soc.

[30] Baik HW, Russell RM (1999). Vitamin B12 deficiency in the elderly. Annu Rev Nutr.

[31] Lupoli R, Lembo E, Saldalamacchia G, Avola CK, Angrisani L, Capaldo B (2017). Bariatric surgery and long-term nutritional issues. World J Diabetes.

[32] Langan RC, Goodbred AJ (2017). Vitamin B12 deficiency: Recognition and management. Am Fam Physician.

[33] Murphy MM, Molloy AM, Ueland PM, Fernandez-Ballart JD, Schneede J, Arija V, Scott JM (2007). Longitudinal study of the effect of pregnancy on maternal and fetal cobalamin status in healthy women and their offspring. J Nutr.

[34] Milman N, Byg KE, Bergholt T, Eriksen L, Hvas AM (2006). Cobalamin status during normal pregnancy and postpartum: a longitudinal study comprising 406 Danish women. Eur J Haematol.

[35] Devi A, Rush E, Harper M, Venn B (2018). Vitamin B12 status of various ethnic groups living in New Zealand: an analysis of the adult nutrition survey 2008/2009. Nutrients.

[36] Gupta AK, Damji A, Uppaluri A (2004). Vitamin B12 deficiency. Prevalence among south asians at a Toronto clinic. Can Fam Physician.

[37] Hashem MM, Esmael A, Nassar AK, El-Sherif M (2021). The relationship between exacerbated diabetic peripheral neuropathy and metformin treatment in type 2 diabetes mellitus. Sci Rep.

[38] Mao-Ying QL, Kavelaars A, Krukowski K (2014). The anti-diabetic drug metformin protects against chemotherapy-induced peripheral neuropathy in a mouse model. PLoS One.

[39] Patil SP, Jain PD, Ghumatkar PJ, Tambe R, Sathaye S (2014). Neuroprotective effect of metformin in MPTP-induced Parkinson's disease in mice. Neuroscience.

[40] Du Y, Zhu YJ, Zhou YX, Ding J, Liu JY (2022). Metformin in therapeutic applications in human diseases: its mechanism of action and clinical study. Mol Biomed.

[41] Davidson JA, Scheen AJ, Howlett HC (2004). Tolerability profile of metformin/glibenclamide combination tablets (Glucovance): a new treatment for the management of type 2 diabetes mellitus. Drug Saf.

[42] (1998). Effect of intensive blood-glucose control with metformin on complications in overweight patients with type 2 diabetes (UKPDS 34). UK Prospective Diabetes Study (UKPDS) Group. Lancet.

[43] Tang T, Lord JM, Norman RJ, Yasmin E, Balen AH (2012). Insulin-sensitising drugs (metformin, rosiglitazone, pioglitazone, D-chiro-inositol) for women with polycystic ovary syndrome, oligo amenorrhoea and subfertility. Cochrane Database Syst Rev.

[44] Campbell JM, Bellman SM, Stephenson MD, Lisy K (2017). Metformin reduces all-cause mortality and diseases of ageing independent of its effect on diabetes control: A systematic review and meta-analysis. Ageing Res Rev.

[45] Hosono K, Endo H, Takahashi H (2010). Metformin suppresses colorectal aberrant crypt foci in a short-term clinical trial. Cancer Prev Res (Phila).

[46] Tan BX, Yao WX, Ge J (2011). Prognostic influence of metformin as first-line chemotherapy for advanced nonsmall cell lung cancer in patients with type 2 diabetes. Cancer.

[47] Dawed AY, Zhou K, van Leeuwen N (2019). Variation in the plasma membrane monoamine transporter (PMAT) (encoded by SLC29A4) and organic cation transporter 1 (OCT1) (encoded by SLC22A1) and gastrointestinal intolerance to metformin in type 2 diabetes: an IMI direct study. Diabetes Care.

[48] Shipton MJ, Thachil J (2015). Vitamin B12 deficiency-a 21st century perspective. Clin Med (Lond).

[49] Lewis CA, de Jersey S, Seymour M, Hopkins G, Hickman I, Osland E (2020). Iron, vitamin B12, folate and copper deficiency after bariatric surgery and the impact on anaemia: a systematic review. Obes Surg.

[50] Carmel R (2008). How I treat cobalamin (vitamin B12) deficiency. Blood.

[51] Oh R, Brown DL (2003). Vitamin B12 deficiency. Am Fam Physician.

[52] Black MM (2008). Effects of vitamin B12 and folate deficiency on brain development in children. Food Nutr Bull.

[53] Higginbottom MC, Sweetman L, Nyhan WL (1978). A syndrome of methylmalonic aciduria, homocystinuria, megaloblastic anemia and neurologic abnormalities in a vitamin B12-deficient breast-fed infant of a strict vegetarian. N Engl J Med.

[54] Devalia V, Hamilton MS, Molloy AM (2014). Guidelines for the diagnosis and treatment of cobalamin and folate disorders. Br J Haematol.

[55] Ahmed MA (2016). Metformin and vitamin B12 deficiency: where do we stand?. J Pharm Pharm Sci.

